# Methionine, threonine and glutamic acid adapted pathways in captive cheetahs on a glycine-supplemented diet

**DOI:** 10.1007/s11306-025-02243-1

**Published:** 2025-04-23

**Authors:** Kathryn M. van Boom, Tertius A. Kohn, Adrian S. W. Tordiffe

**Affiliations:** 1 Department of Paraclinical Sciences, Faculty of Veterinary Science, University of Pretoria, Onderstepoort, South Africa; 2 Department of Medical Bioscience, Faculty of Natural Sciences, University of the Western Cape, Bellville, South Africa; 3 Centre for Veterinary Wildlife Studies, Faculty of Veterinary Science, University of Pretoria, Onderstepoort, South Africa

**Keywords:** *Acinonyx jubatus*, Amino acids, Cheetah, Metabolomics, NMR, Nutrition

## Abstract

**Background:**

Captive cheetahs are prone to a range of unusual diseases potentially linked to unnatural diets high in muscle meat and low in collagen-rich animal fibre. In the wild, cheetahs typically eat whole prey diets not easily replicated in a captive setting. Glycine is the most abundant amino acid in collagen with a key role in several metabolic pathways such as collagen biosynthesis. Several recent studies suggest that endogenous glycine production may be limited in several species.

**Objectives:**

Using untargeted ^1^H- nuclear magnetic resonance, the metabolic changes in the urine and serum of 10 adult captive cheetahs on a glycine-supplemented diet were investigated.

**Methods:**

Cheetahs were fed either a meat only (control) or glycine-supplemented meat diet (30 g glycine per 1 kg meat) for four weeks, followed by a four-week cross-over. Urine and blood samples were collected at baseline and after each intervention.

**Results:**

A total of 151 and 60 metabolites were identified in the urine and serum, respectively. Specifically, dimethylsulphone, proline, fructose, dimethylamine, trimethylamine, pyroglutamic acid, 1,3-diaminopropane, dihydrothymine, methylmalonic acid and pimelic acid contributed to metabolome differences in the urine. In serum, glutamic acid, threonine, α-aminobutyric acid, glucose-6-phosphate, ethanolamine, methionine and propionic acid were highlighted. These metabolites play various metabolic roles in energy production, immune function, protein and collagen biosynthesis or as products of gut microbiome fermentation.

**Conclusion:**

Glycine supplementation influenced threonine sparing, pyrimidine biosynthesis pathways and bacterial fermentation products, although the implications of these findings on the health of captive cheetahs is unknown. Future studies should use a targeted approach to further elaborate on these pathways.

**Supplementary Information:**

The online version contains supplementary material available at 10.1007/s11306-025-02243-1.

## Introduction

Cheetahs (*Acinonyx jubatus)* are a unique and vulnerable felid species, currently only occupying 9% of their global historic range and, therefore, requiring human protection and care as an important tool for their conservation (Durant et al., 2017). However, in captivity they are prone to an array of diseases that are not observed in their free ranging counterparts such as chronic lymphoplasmacytic gastritis, glomerulosclerosis and veno-occlusive disease (Munson, 1993; Munson et al., 1999). Various factors of captivity, namely stress, lack of physical activity and diet have been suggested as possible causes of the increased disease prevalence. To date, no studies have investigated the link between physical activity and disease in cheetahs and the evidence regarding the effects of stress is somewhat inconsistent. In captivity, cheetahs primarily consume raw muscle rich meat diets in contrast to the wild, whole carcass diets with collagen-rich components like skin, cartilage and bone (Depauw et al., 2013; Whitehouse-Tedd et al., 2015). In rats, the digestibility of collagen (in the form of pig skin) is well over 90% due to the breakdown by pepsin and hydrochloric acid in the stomach, as well as by trypsin and chymotrypsin from the pancreas (Reuterswärd & Fabiansson, 1985) and this is likely similar in carnivores that produce large amounts of gastric acid and pepsin. Although carcass components are often provided in captivity, the consistent feeding of this diet may not always be feasible due to funding or logistical challenges. Certain aspects of this high protein raw muscle meat diet may be contributing to the high disease rate– these include incomplete protein digestion leading to increased production of phenolic compounds by gut bacteria, reduced nutrient diversity and increased gut bacterial substrate fermentation (Depauw et al., 2012, 2013; Vester et al., 2008).

Recently, the effect of dietary glycine supplementation was investigated in 10 captive cheetahs and resulted in decreased body weight, albumin, alkaline phosphatase and total calcium concentration and an increased eosinophil and basophil count (van Boom et al., 2024). Glycine, the simplest amino acid, has an array of physiological functions including the synthesis of collagen, a vital structural protein in all animals (Li & Wu, 2018). Additionally, glycine is important in the conjugation and subsequent excretion of several detrimental bacterial by-products of protein digestion including ammonia, phenolic and indolic compounds (Badenhorst et al., 2014; Depauw et al., 2013). Cheetahs excrete large amounts of phenylalanine, tyrosine and glycine conjugated organic acids that indicate a substantial metabolic need for glycine to aid in the digestion of their high protein diets (Tordiffe et al., 2017). Conventionally glycine and taurine are involved in digestion and absorption through the conjugation of bile acids, but only taurine has been found to conjugate bile acids in domestic cats — whether this is also true for cheetahs is unknown (MacDonald et al., 1984; Wang et al., 2013). Therefore, further understanding the metabolic role of collagen and glycine through more comprehensive analytical techniques may provide essential information on the effects of the captive diet. One such metabolomics technique is nuclear magnetic resonance (NMR) which provides structural information of small organic molecules based on atom-centred nuclear interactions and, although it is less sensitive than mass spectrometry methods, is considered a robust, reliable and reproducible technique (Lenz & Wilson, 2007).

The majority of metabolomics studies have been conducted in domestic or companion animals, with limited studies in wild or captive animals. Only a handful of studies have been conducted using metabolomic methods on biological fluids of captive or free-roaming cheetahs to investigate metabolic properties (Tordiffe et al., 2016, 2017; Tordiffe & Mienie, 2019). Tordiffe et al. (2017) detected 339 organic acids in the urine of captive cheetahs, of which, phenolic compounds linked to the anaerobic fermentation of aromatic amino acids in the intestine and glycine conjugates were present in high concentrations - potentially linked to their high muscle meat intake and the bacterial fermentation thereof. Furthermore, Tordiffe and Mienie (2019) identified 38 urinary and 36 serum amino acids of which arginine and glutamine were the most abundant, respectively. Cystine had the highest fractional excretion indicating that a high percentage of cystine is excreted and not reabsorbed by the body. This may be due to exogenous methionine and serine from the consumed animal tissue meeting the high metabolic demand of glutathione, taurine, CoA and general protein synthesis.

Glycine has many physiological functions and, as a result, there are many glycine linked metabolites found in urine and serum, most notably glycine conjugation substrates produced by gut microorganisms (Badenhorst et al., 2014). The amount of glycine conjugates excreted in urine depends on the dietary intake of polyphenolic compounds and the extent of their fermentation in the colon. In cheetahs, the increased production of phenolic compounds would potentially be through the fermentation of aromatic amino acids by gut bacteria. Additionally, glycine forms part of the biosynthesis of carnitine from methionine and lysine (as an intermediary product) and creatine which can also be excreted in urine, the latter as creatinine. Glutathione and benzoate metabolism are indicators of glycine deficiency, with increased excretion of pyroglutamic acid and hippurate acting as markers, respectively (Meléndez-Hevia et al., 2009). In humans, pigs and other mammals the amount of endogenous glycine synthesised does not meet the metabolic demand and, therefore, additional glycine should be obtained either from the diet or through supplementation (Meléndez-Hevia et al., 2009; Wang et al., 2013).

Considering its diverse metabolic role and the abundance of glycine conjugates found in cheetah urine, glycine is likely required in significant amounts in the cheetah (Tordiffe et al., 2017). Yet it is potentially deficient in the high-protein captive diet due to the reduced intake of collagen-rich carcass components. Despite its endogenous production from other amino acids such as serine and threonine, these pathways may be severely constrained (Meléndez-Hevia et al., 2009). Therefore, understanding the metabolic role of collagen and glycine in cheetahs may provide novel information on the effects of the captive diet. The use of increasingly popular metabolomics techniques in veterinary and nutritional studies, may be a useful tool to investigate these effects. This approach has not previously been used to investigate the effect of diet in captive cheetahs or any other exotic felid. Therefore, the aim of this study was to investigate the metabolic differences between glycine supplemented and non-supplemented captive cheetahs in a randomised cross-over study design. This was achieved through an untargeted metabolomics approach using NMR analysis on urine and serum samples in 10 captive cheetahs. The current study is an extension of the work previously reported by van Boom et al. (2024).

## Method

### Study design, animals and feeding intervention

The randomised cross-over study design and feeding intervention utilised in the current study as well as the animal characteristics have previously been described in detail (van Boom et al., 2024). Briefly, 10 cheetahs housed at a conservation facility (Cango Wildlife Ranch in Oudtshoorn, Western Cape, South Africa) were included in the study. They had an average age of 4.2 ± 1.3 (males; *n* = 5) and 3.2 ± 1.1 (females; *n* = 5) years and were healthy with no clinical signs of gastritis or renal failure. Ethical approval for this study was obtained from the Animal Research Ethics Committees of the Faculty of Veterinary Science, University of Pretoria (REC231-19) and the University of the Western Cape (AR20/3/4). A standing Threatened or Protected Species (TOPS) permit for the Faculty of Veterinary Science (reference number: S02559) and a Department of Agriculture, Forestry’s and Fisheries Sect. 20 permit (reference number: 12/11/1/7) were also obtained for this project.

A three-week habituation period was implemented prior to the start of the study to standardise the varied diets across all 10 cheetahs. During this time, cheetahs were fed six days a week with a control diet of only horse muscle mince with 10 g of vitamin and mineral supplement added for every 1 kg mince (Panthera Supplement, WildCat Nutrition, Pretoria, South Africa). The detailed nutritional breakdown of horse muscle meat and the vitamin and mineral supplement are shown in Supplementary Table S1 and have previously been described (Badiani et al., 1997; van Boom et al., 2024). Briefly, a 100 g muscle meat contains approximately 19.8 g protein: 8.8 g are essential amino acids (leucine and lysine contributing ≈ 1.5 g each; and histidine, isoleucine and valine contributing ≈ 1 g each), 9.8 g are conditionally essential amino acids (2.8 g glutamic acid, and alanine, arginine, aspartic acid and glycine contributing over 1 g), 0.2 g hydroxyproline and 1.2 g total collagen. The habituation was implemented to control the glycine intake of the cheetahs since collagen (from skin and bones) contains high concentrations of glycine, but glycine is relatively low in muscle meat as characterised by the control diet. Baseline data and samples were collected after the three-week habituation period.

Cheetahs were then randomly assigned to a control (*n* = 6) or glycine-supplemented group (*n* = 4). Those on the control diet continued with the habituation diet, whereas the cheetahs in the glycine group were fed an additional 30 g of glycine powder per 1 kg meat (Glycine, WildCat Nutrition, Pretoria, South Africa). Cheetahs remained on these diets for four weeks, with one fasting day per week, whereafter the second sample collection occurred. All animals then underwent a two-week washout period where they were fed the control diet. The groups were then crossed over and fed the opposite diet for another four weeks, after which the third sample collection occurred.

### Immobilisation

The cheetahs were immobilised at each sampling event via intramuscular hand injection with 30 µg/kg medetomidine hydrochloride (10 mg/ml, Medetomidine, Kyron Laboratories Pty LTD, South Africa) in combination with 1 mg/kg zolazepam/tiletamine (100 mg/ml, Zoletil ^®^, Virbac, South Africa). Cheetahs were maintained under anaesthesia with 1–2% isoflurane in oxygen for the one-hour collection period. Once all the samples were collected, the sedation was reversed with 5 mg intramuscular atipamezole (5 mg/ml, Antisedan^®^, Pfizer, South Africa). The standard anaesthetic and cardiovascular parameters of the cheetahs were monitored while they were under sedation and after reversal.

### Serum and urine collection and storage

Blood was collected from the jugular vein, using an 18G needle and a 20 ml syringe, immediately transferred to a serum tube where it was allowed to clot for 30 min at room temperature and subsequently centrifuged for 10 min at 6000 rpm. The supernatant was transferred to a new microtube and stored at −20 °C.

Urine, collected via urethral catheterisation using a sterile 6FG 120 mm feeding tube, was centrifuged for 10 min at 6000 rpm to remove any cellular debris, transferred to a microtube and stored at −20 °C.

### NMR analysis

An untargeted whole metabolome ^1^H-NMR approach was utilised to identify and quantify the metabolites present in the urine and serum samples.

#### Urine sample preparation

A 1 ml urine aliquot was added to a micro tube and centrifuged for 5 min at 12 000 x g. Thereafter, 540 µl was transferred to another microtube and mixed with 60 µl of NMR buffer solution (9:1). The NMR buffer solution consisted of 1.5 M potassium phosphate buffer (pH 7.4) in deuterium oxide and 5.8 mM trimethylsilyl-2,2,3,3-tetradeuteropropionic acid (TSP; Sigma-Aldrich, St. Louis, Missouri, USA) with a trace amount of sodium azide included to prevent bacterial growth in the sample. This was followed by centrifugation for 5 min at 12 000 x g. A final volume of 540 µl was transferred to a 5 mm NMR tube for metabolomics analysis.

#### Serum sample preparation

Samples were filtered using Amicon Ultra 2 ml centrifugal units with 10 kDa membrane filters (Merck; UFC201024). Prior to use, each centrifugal filter unit was pre-rinsed with dH_2_0 and centrifuged at 4500 x g for 10 min to remove trace amounts of glycerol and glycerine from membrane filters, which can interfere with NMR signals. Subsequently, 800 µl serum was filtered, centrifuged at 4500 x g for 30 min, after which 540 µl of each filtrate was combined with 60 µl buffer solution (same as urine). The sample mixture was centrifuged at 12 000 x g for 5 min, whereafter 540 µl was transferred to a 5 mm NMR tube for metabolomics analyses.

#### System and experimental settings

The same settings were used for urine and serum analyses. A 500 MHz Bruker Avance III HD NMR spectrometer equipped with a triple-resonance inverse (TXI) ^1^H {^15^N, ^13^C} probe head and x, y, z gradient coils was used to analyse the samples. H spectra were acquired as 128 transients in 32k data points with a spectral width of 10,504Hz and acquisition time of 3.12s. The receiver gain was set to 90.5. The sample temperature was maintained at 300K and the H_2_O resonance was pre-saturated by single-frequency irradiation during a relaxation delay of 4s, with a 90 excitation pulse of 8 s. Shimming of the sample was performed automatically on the deuterium signal. Fourier transformation and phase and baseline correction were done automatically. Software used for NMR processing was Bruker Topspin (version 3.5).

### Data analysis

The raw Bruker Topspin spectral data files were analysed using R programming software (R, version 4.0.4) and RStudio integrative environment (RStudio, version 1.4.1106, MA, USA). The Automatic Statistical Identification in Complex Spectra (ASICS; version 2.6.1, Bioconductor, NY, USA) R package was used to identify and quantify metabolites in the complex spectra (detailed in Lefort et al. (2019)). The following sections are a summary of the various data analysis steps.

#### Data processing

The raw Bruker spectral files were imported into RStudio and the complex spectrum underwent several pre-processing steps. Firstly, baseline distortions caused by instrument instability were corrected by constructing a baseline and removing it from the spectrum. Secondly, a speaq algorithm was used to align the spectra against an automatically detected reference spectrum to remove any metabolite peak position change due to temperature or pH variations. Thirdly, unwanted regions in the spectrum corresponding to water (4.5 to 5.1 ppm) and urea (5.5 to 6.5 ppm, for urine) were removed to reduce interference. The final pre-processing step was normalisation, which minimises the systematic variations due to differences in sample dilutions, therefore making samples comparable - the Probabilistic Quotient Normalisation (PQN) method was used for this dataset (Dieterle et al., 2006). This method was implemented instead of correcting to creatinine concentrations because creatinine has been shown to be highly variable in cheetahs (Tordiffe et al., 2017). The ASICS package provides a pre-processed library of the spectra of pure compounds which was used as a reference to identify and quantify metabolites.

#### Metabolite identification and quantification

The identification of metabolites in the complex sample mixture relies on various linear and warping functions. A least squares method was used to quantify the identified metabolites. This did not allow identification of metabolites at an absolute concentration below 1 µM, as the signal to noise ratio was too low, as well as metabolites at an absolute concentration above 1 M which resulted in broadening and overlapping of the NMR signals. This process identified metabolites and provided an estimated relative concentration reflecting a function of the complex mixture, reference library and number of protons for each selected metabolite.

### Statistical analysis

All statistical analysis for NMR data was conducted using MetaboAnalyst version 5.0 (https://www.metaboanalyst.ca) (Pang et al., 2021). JMP Pro version 16.1.0 (SAS Institute, North Carolina, USA) was used to generate the figures. Data are expressed as mean ± standard deviation where appropriate and significance was set at *p* < 0.05.

The current study design utilised two uneven groups that experienced slightly varied lengths on each diet as well as either one or two dietary changes during the study. As a result, it was necessary to first check the urine and serum metabolite analysis for each group before examining the combined effect. The steps described below were conducted for both the *n* = 6 group and the combined *n* = 10 group. After the analysis, it was deemed suitable to use the combined *n* = 10 groups as it produced more consistent data.

In order to obtain a more holistic statistical overview, multiple univariate and multivariate tests were utilised after the data normalisation steps. The various approaches are described below, which combined, give rise to the notable potentially important metabolites of interest.

#### Data cleaning and scaling

Metabolites that were present in less than 50% of the samples in all three groups were removed in order to reduce the effect of infrequently observed metabolites across all samples. After cleaning, the metabolite concentration list was imported into MetaboAnalyst 5.0. The data underwent various steps prior to analysis, including an integrity check, replacing zero values by 20% of the lowest value, data filtering based on interquartile range (removing approximately 10% of outlying values) and auto scaling normalisation (to the sample mean and standard deviation to allow better metabolite comparison). As there was a wide range of concentrations between metabolites, these steps were necessary to ensure that all the values were equally weighted especially for multivariate analysis. It makes use of the following equation to calculate the scaled value for a single metabolite of a sample: scaled value = (X_2_- X_1_)/SD_1_ where X_2_ is an individual sample concentration, X_1_ is the mean for all samples, SD_1_ is the standard deviation for all samples.

#### Univariate and multivariate analyses

A one-way repeated measures ANOVA was performed to identify differences in metabolites between groups. False Discovery Rate (FDR) was used to correct for the multiple univariate comparisons and protect against false positive results. The normalised and scaled data of the significantly identified metabolites from MetaboAnalyst was imported into JMP and a Tukey-Kramer HSD post hoc analysis performed.

The unsupervised principal component analysis (PCA) and the supervised partial least squares discriminant analysis (PLS-DA) data reduction methods were performed on the normalised data in MetaboAnalyst. Loading scores, which indicate the influence of each metabolite on the data variation, were recorded for PCA. For PLS-DA, the leave-one-out cross validation method was used for five components in which the cross validated sum of the squares (Q2) was an estimate of the predictive ability of the model. The variable importance in projection (VIP) and the weighted sum of absolute coefficient of regression for each diet, were recorded for each metabolite.

#### Identifying metabolites of interest

The metabolites identified with the one-way ANOVA and the top 20% of metabolites with the greatest influence under each parameter of the PCA (loading scores) and PLS-DA (VIP and coefficient of regression) analysis were highlighted. These metabolites were further reduced to those that were present in four or more analyses out of a potential of eight. This ultimately created a list of potentially important urinary and serum metabolites in cheetahs on a baseline, control and glycine-supplemented diet.

## Results

### Identified urinary metabolites and relative concentration

A total of 151 metabolites across all 30 urine samples were identified. After data cleaning, 25% of the metabolites were removed and 112 metabolites remained. The most abundant metabolites for each group and their relative concentrations are indicated in Table 1. Creatinine was the most abundant with a five to six times higher relative concentration than allantoin, the next abundant metabolite. L-cysteine and malonic acid were also abundant across all three groups, while L-ornithine (baseline), TMAO (control) and glycolic acid (glycine diet) were abundant in the respective diets.

**Table 1:** Abundant urinary metabolites in cheetahs at baseline, on a control diet and on a glycine-supplemented diet (*n* = 10)

Baseline			Control diet			Glycine-supplemented diet		
Metabolite	*n*	Relative concentration	Metabolite	*n*	Relative concentration	Metabolite	*n*	Relative concentration
Creatinine	10	64.1 ± 47.4	Creatinine	10	72.8 ± 37.6	Creatinine	10	56.9 ± 46.2
Allantoin	10	14.3 ± 3.2	Allantoin	10	13.7 ± 2.9	Allantoin	10	15.2 ± 3.8
L-Cysteine	10	10.5 ± 0.7	Malonic acid	10	13.2 ± 4.9	L-Cysteine	10	10.1 ± 1.6
Malonic acid	10	7.8 ± 4.7	TMAO	10	10.2 ± 7.2	Glycolic acid*	7	9.6 ± 6.2
L-Ornithine	10	6.1 ± 1.3	L-Cysteine	10	9.9 ± 0.9	Malonic acid	10	9.6 ± 4.0
L-Proline	10	4.5 ± 0.4	L-Carnitine	10	7.3 ± 2.6	TMAO	10	7.3 ± 4.7
DHA	10	4.3 ± 1.1	L-Ornithine	10	5.8 ± 0.9	Guanidinoacetic acid*	7	6.8 ± 5.2
L-Carnitine	10	3.9 ± 2.3	L-Proline	10	5.5 ± 0.7	L-Ornithine	10	6.4 ± 1.2
TMAO	10	3.9 ± 3.6	Taurine	10	4.4 ± 1.4	L-Proline	10	5.1 ± 0.5
1,3-DAP	10	3.4 ± 0.3	DHA	9	3.9 ± 0.9	L-Carnitine	10	4.9 ± 2.2
L-Tryptophan	8	3.1 ± 0.6	Choline chloride	10	3.8 ± 1.2	DHA	10	4.1 ± 0.8
L-Arginine	10	3.1 ± 0.5	Phosphocholine	10	3.5 ± 0.9	Taurine	10	3.8 ± 1.1
Taurine	10	3.0 ± 1.1	α-Ketoglutaric acid	10	3.4 ± 0.4	L-Glutamine	10	3.2 ± 0.8
Choline chloride	10	3.0 ± 1.1	L-Glutamine	10	3.4 ± 1.1	Choline chloride	10	3.2 ± 1.1
α-Ketoglutaric acid	10	2.7 ± 0.4	D-Glucose	10	3.2 ± 0.6	1,3-DAP	10	3.1 ± 0.3
D-Maltose	10	2.6 ± 0.4	D-Maltose	10	3.2 ± 0.5	L-Arginine	10	3.0 ± 0.3
D-Glucose	10	2.6 ± 0.4	L-Arginine	10	3.2 ± 0.7	α-Ketoglutaric acid	10	2.9 ± 0.3
L-Lysine	9	2.6 ± 0.4	Dimethylglycine	10	3.2 ± 2.1	L-Tryptophan	10	2.8 ± 0.6
Phosphocholine	10	2.6 ± 1.0	Creatine	7	3.1 ± 2.8	Phosphocholine	10	2.8 ± 0.7
L-Citrulline	10	2.6 ± 0.2	1,3-DAP	10	2.9 ± 0.3	D-Maltose	10	2.6 ± 0.6

Data are expressed as mean ± SD and determined based on the number of samples (n) where the metabolite was detected. Only those present in > 5 samples were included in the table. * indicates metabolites that were present at high relative concentration in < 5 samples of the baseline and control diets. Relative concentration is an arbitrary unit. TMAO, Trimethylamine oxide; 1,3-DAP, 1,3-Diaminopropane; DHA, dehydroascorbic acid

### Urinary metabolites identified by univariate analysis

Following post hoc analysis, 27 metabolites were significantly different between groups (Table 2). Dimethylamine (DMA) had the greatest significance with increased concentrations on the control and glycine-supplemented diets. The relative concentrations of the control diet were greater than at baseline for 22 of the identified metabolites, with only 1,3-diaminopropane having a higher concentration at baseline compared to the control diet. Similarly, the relative concentrations of the control diet were higher than the glycine-supplemented diet for 11 metabolites with the glycine diet being greater than the control for fructose and CMP. For most of the metabolites, the baseline and glycine-supplemented diets were similar with the exceptions being DMA and methylmalonic acid.

**Table 2:** Significant metabolites in the urine of cheetahs at baseline, on a control diet and glycine-supplemented diet (*n* = 10) based on univariate analysis

Metabolite	F-value	Adjusted *p*-value	Tukey-Kramer *p*-value		
			Baseline-Control	Baseline-Glycine	Control-Glycine
Dimethylamine	28.56	0.00	**< 0.01** (C > B)	**0.01** (G > B)	NS
α-Ketoglutaric acid	19.64	0.00	**< 0.01** (C > B)	NS	**0.02** (C > G)
Methylguanidine	15.91	0.00	**< 0.01** (C > B)	NS	**< 0.01** (C > G)
L-Carnitine	14.86	0.00	**0.01** (C > B)	NS	NS
Dimethylsulphone	14.53	0.00	**< 0.01** (C > B)	NS	**< 0.01** (C > G)
L-Proline	12.57	0.01	**< 0.01** (C > B)	NS	NS
L-Cystine	12.35	0.01	**0.05** (C > B)	NS	NS
Pyroglutamic acid	11.95	0.01	**< 0.01** (C > B)	NS	NS
Trimethylamine	10.96	0.01	**< 0.01** (C > B)	NS	**< 0.01** (C > G)
Methylmalonic acid	10.89	0.01	NS	**0.04** (B > G)	NS
L-Asparagine	10.30	0.01	**0.02** (C > B)	NS	**0.01** (C > G)
Trans-Aconitic acid	9.71	0.01	**0.02** (C > B)	NS	NS
L-Carnosine	9.51	0.01	**< 0.01** (C > B)	NS	NS
D-Fructose	9.31	0.01	NS	NS	**< 0.01** (G > C)
D-Maltose	9.19	0.01	**0.03** (C > B)	NS	**0.02** (C > G)
Taurine	8.32	0.02	**0.04** (C > B)	NS	NS
Malonic acid	7.25	0.03	**0.04** (C > B)	NS	NS
β-Alanine	6.94	0.03	**0.03** (C > B)	NS	NS
Acetoacetic acid	6.84	0.03	**0.01** (C > B)	NS	NS
L-Valine	6.53	0.04	**0.04** (C > B)	NS	**0.01** (C > G)
3-Hydroxyphenylacetic acid	6.52	0.04	NS	NS	**0.04** (C > G)
Dimethylglycine	6.17	0.04	**< 0.01** (C > B)	NS	**< 0.01** (C > G)
D-Glucose	5.72	0.05	**0.03** (C > B)	NS	**0.02** (C > G)
1-Methylhydantoin	5.67	0.05	**< 0.01** (C > B)	NS	**0.02** (C > G)
CMP	5.49	0.05	NS	NS	**0.04** (G > C)
TMAO	5.46	0.05	**0.04** (C > B)	NS	NS
1,3-DAP	5.40	0.05	**0.04** (B > C)	NS	NS

Analysis was performed on normalised data. NS,* not significant; B*,* baseline; C*,* control diet; G*,* glycine diet; CMP*, cytidine monophosphate; *TMAO*,* Trimethylamine oxide; 1*,*3-DAP*,* 1*,*3-Diaminopropane*

### Urinary metabolites identified by PCA and PLS-DA analysis

**Fig. 1:**
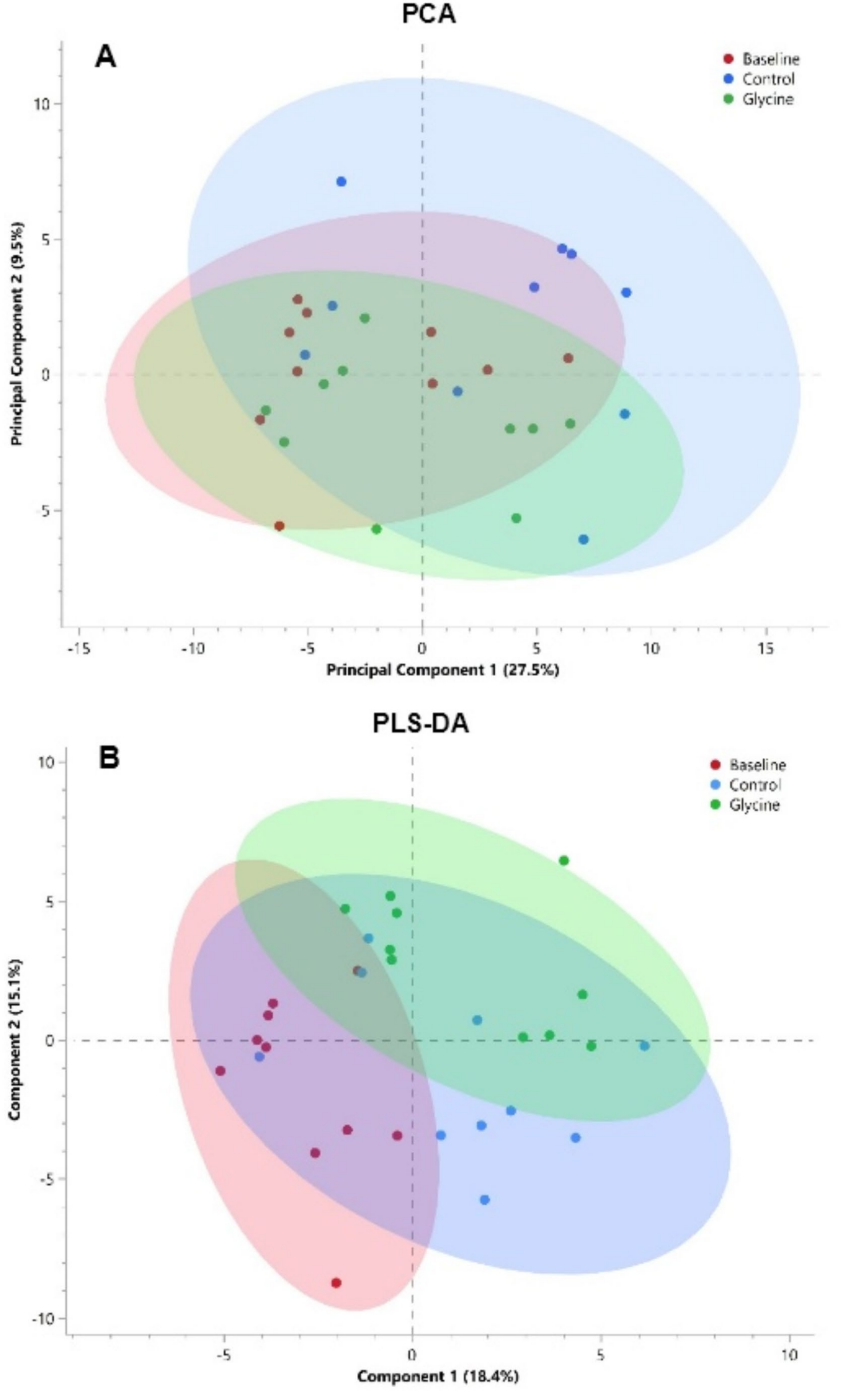
A two-dimensional **A** PCA and **B** PLS-DA scores plot of urinary metabolites of 10 cheetahs at baseline, on the control and glycine-supplemented diets. Each point represents an individual cheetah at baseline (red), control (blue) and glycine supplemented (green) with a corresponding 95% confidence ellipse for each diet

The PCA and PLS-DA score plots are shown in Fig. 1. PC 1 accounts for 27.5% of the variation seen across the data, while PC 2 accounts for 9.5% of the variation. The loadings or contribution of the top 10 metabolites of PC 1 and PC 2 are shown in Supplementary Table S2. The separation seen on the PLS-DA plot (Fig. 1B) between the diets is noticeably small. The discriminant variables of component 1 accounts for 18.4% of the variation between the diets, while component 2 accounts for 15.1% of the variation observed between diets. The difference between the glycine-supplemented and baseline diets account for the greatest separation along component 1 and component 2, respectively. The overall VIP scores and the coefficient variables of each diet for the first 10 metabolites are shown in Supplementary Table S2.

### Identified serum metabolites and relative concentration

A total of 60 metabolites, which was further reduced to 48, were identified in the serum across the 10 cheetahs in each diet. The relative concentrations (arbitrary units) of the most abundant metabolites are reported in Table 3. D-glucose was the most abundant metabolite identified across all three groups; followed by L-glutamine, lactic acid, taurine and guanidinoacetic acid.

**Table 3:** Abundant serum metabolites of cheetahs at baseline, on a control diet and on a glycine-supplemented diet (*n* = 10)

Baseline			Control diet			Glycine-supplemented diet		
Metabolite	*n*	Relative concentration	Metabolite	*n*	Relative concentration	Metabolite	*n*	Relative concentration
**D-Glucose**	10	23.9 ± 2.9	**D-Glucose**	10	25.0 ± 5.4	**D-Glucose**	10	23.4 ± 3.0
**L-Glutamine**	10	4.9 ± 0.6	**L-Glutamine**	10	4.9 ± 0.5	**L-Glutamine**	10	4.7 ± 0.4
**Lactic acid**	10	3.5 ± 0.6	**Lactic acid**	10	3.7 ± 0.4	**Lactic acid**	10	3.8 ± 0.5
**Taurine**	10	2.7 ± 1.0	**Taurine**	10	3.5 ± 0.9	**Taurine**	10	2.9 ± 0.9
**Guanidinoacetic acid**	10	2.7 ± 0.6	**Guanidinoacetic acid**	10	2.9 ± 0.8	**Guanidinoacetic acid**	10	2.6 ± 0.5
**D-Mannose**	10	2.6 ± 0.3	**D-Mannose**	10	2.4 ± 0.3	**G-6-P**	10	2.5 ± 0.2
**G-6-P**	10	2.5 ± 0.3	**D-Glucuronic acid**	10	2.4 ± 0.5	**D-Mannose**	10	2.4 ± 0.3
**Glyceric acid**	10	2.3 ± 0.4	**G-6-P**	10	2.3 ± 0.2	**D-Glucuronic acid**	10	2.2 ± 0.4
**D-Glucuronic acid**	10	2.3 ± 0.4	**Glyceric acid**	9	2.3 ± 0.4	**Glyceric acid**	10	2.2 ± 0.4
**D-Galactose**	9	1.9 ± 0.3	**D-Galactose**	7	2.0 ± 0.3	**D-Galactose**	10	1.8 ± 0.3
**L-Carnitine**	10	1.8 ± 0.4	**L-Alanine**	10	1.7 ± 0.2	**L-Alanine**	10	1.7 ± 0.3
**L-Alanine**	10	1.7 ± 0.2	**L-Carnitine**	10	1.7 ± 0.6	**L-Carnitine**	10	1.6 ± 0.5
**D-Maltose**	10	1.6 ± 0.2	**D-Maltose**	9	1.6 ± 0.2	**D-Maltose**	10	1.6 ± 0.1
**L-Glycine**	10	1.5 ± 0.3	**L-Glycine**	10	1.5 ± 0.5	**L-Cystine**	10	1.5 ± 0.2
**L-Cystine**	10	1.5 ± 0.1	**L-Cystine**	10	1.5 ± 0.2	**L-Glycine**	10	1.4 ± 0.3
**D-Fructose**	7	1.2 ± 0.4	**D-Fructose**	7	1.5 ± 0.4	**L-Proline**	10	1.3 ± 0.2
**L-Proline**	10	1.2 ± 0.2	**Ethanolamine**	10	1.2 ± 0.3	**Ethanolamine**	10	1.2 ± 0.2
**Ethanolamine**	10	1.1 ± 0.1	**L-Proline**	10	1.2 ± 0.3	**Creatinine**	10	1.1 ± 0.2
**Creatinine**	10	1.1 ± 0.2	**L-Lysine**	6	1.1 ± 0.2	**D-Sorbitol**	8	1.1 ± 0.2
**D-Sorbitol**	9	1.0 ± 0.2	**Creatinine**	10	1.1 ± 0.1	**D-Fructose**	6	1.1 ± 0.4

Data are expressed as mean ± SD and determined based on the number of samples (n) where the metabolite was detected. Relative concentration is an arbitrary unit. G-6-P, *D-Glucose-6-Phosphate*

### Serum metabolites identified with univariate, PCA and PLS-DA analysis

**Fig. 2:**
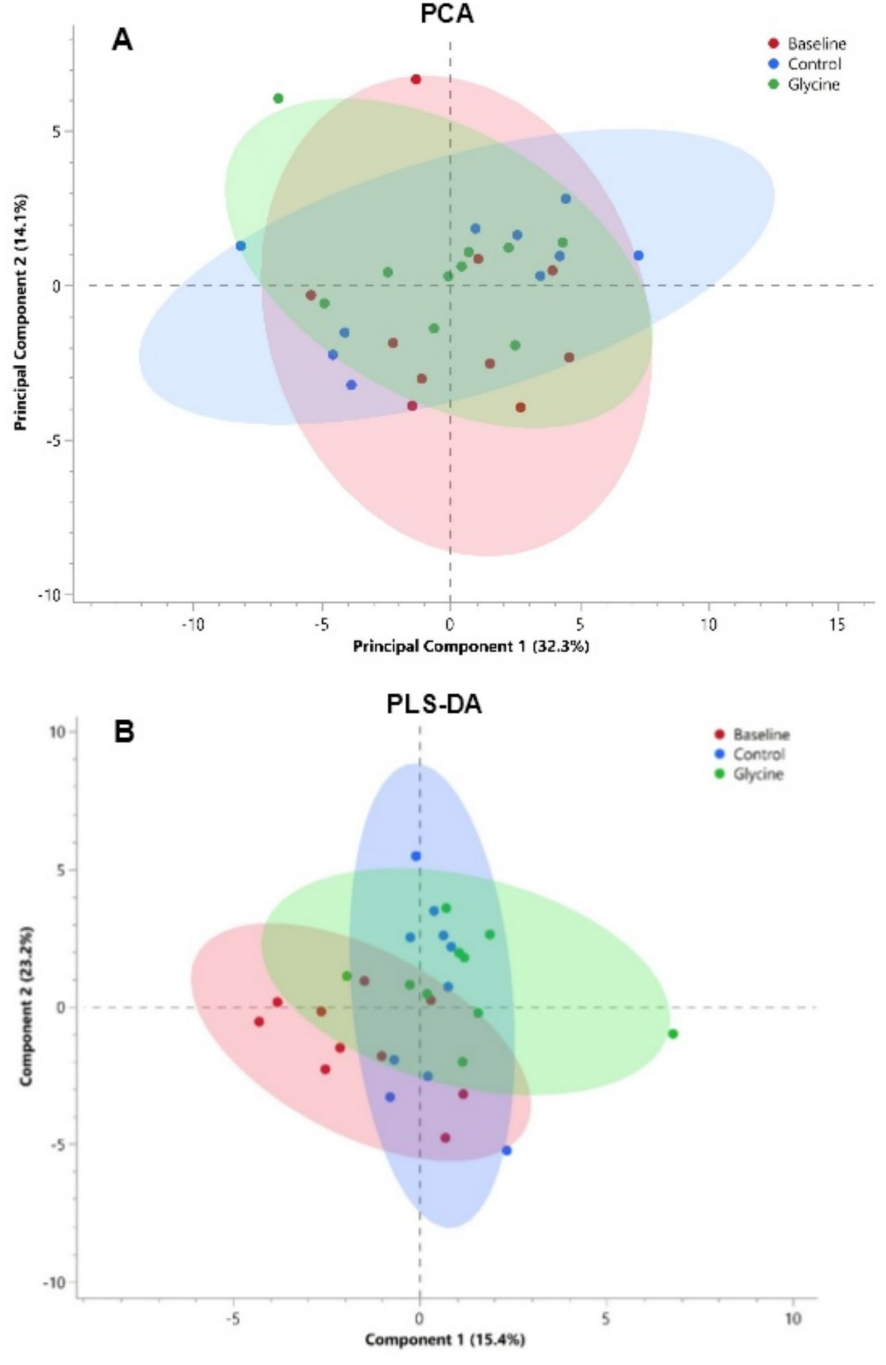
A two-dimensional **A** PCA and **B** PLS-DA scores plot of serum metabolites of 10 cheetahs at baseline, on the control and glycine-supplemented diets. Each point represents an individual cheetah at baseline (red), control (blue) and glycine supplemented (green) with a corresponding 95% confidence ellipse for each diet

L-glutamic acid (F = 6.14, raw *p* = 0.01, adjusted *p* = 0.42) and α-aminobutyric acid (AABA) (F = 3.92, raw *p* = 0.04, adjusted *p* = 0.65) were significant prior to the adjustment, but no metabolites were deemed significant through univariate analysis. The PCA and PLS-DA plots are shown in Fig. 2. PC 1 accounted for 32% variation in the serum data, while PC 2 accounted for 14%. Most of the data for all three diets were clustered, with three clear outliers– the baseline and control diet outliers represent the same individual. The 10 metabolites contributing the most to the variation of PC 1 and PC 2 are reported in Supplementary Table S3. In the PLS-DA plot, there is a clear overlap with the baseline, control and glycine diet. The discriminant variables of component 1 account for 15.4% of the variation between diets, while component 2 is 23.2%. The latter highlights the limited predictive power of this model. The VIP and co-efficient scores for each diet is reported in Supplementary Table S3.

### Important urinary and serum metabolites

Ten urinary metabolites were identified in five or more analyses– dimethylsulphone (DMSO_2_), L-proline, D-fructose, dimethylamine (DMA), trimethylamine (TMA), pyroglutamic acid, 1,3-diaminopropane, dihydrothymine, methylmalonic acid and pimelic acid. Significance was not identified univariately between groups for dihydrothymine and pimelic acid. Interestingly, dihydrothymine was identified more frequently on the glycine diet (*n* = 9) compared to the baseline (*n* = 5) and control (*n* = 6) diets. Similarly, fructose was more frequent on the glycine diet (*n* = 10) compared to baseline (*n* = 8) and control diet (*n* = 3).

**Fig. 3:**
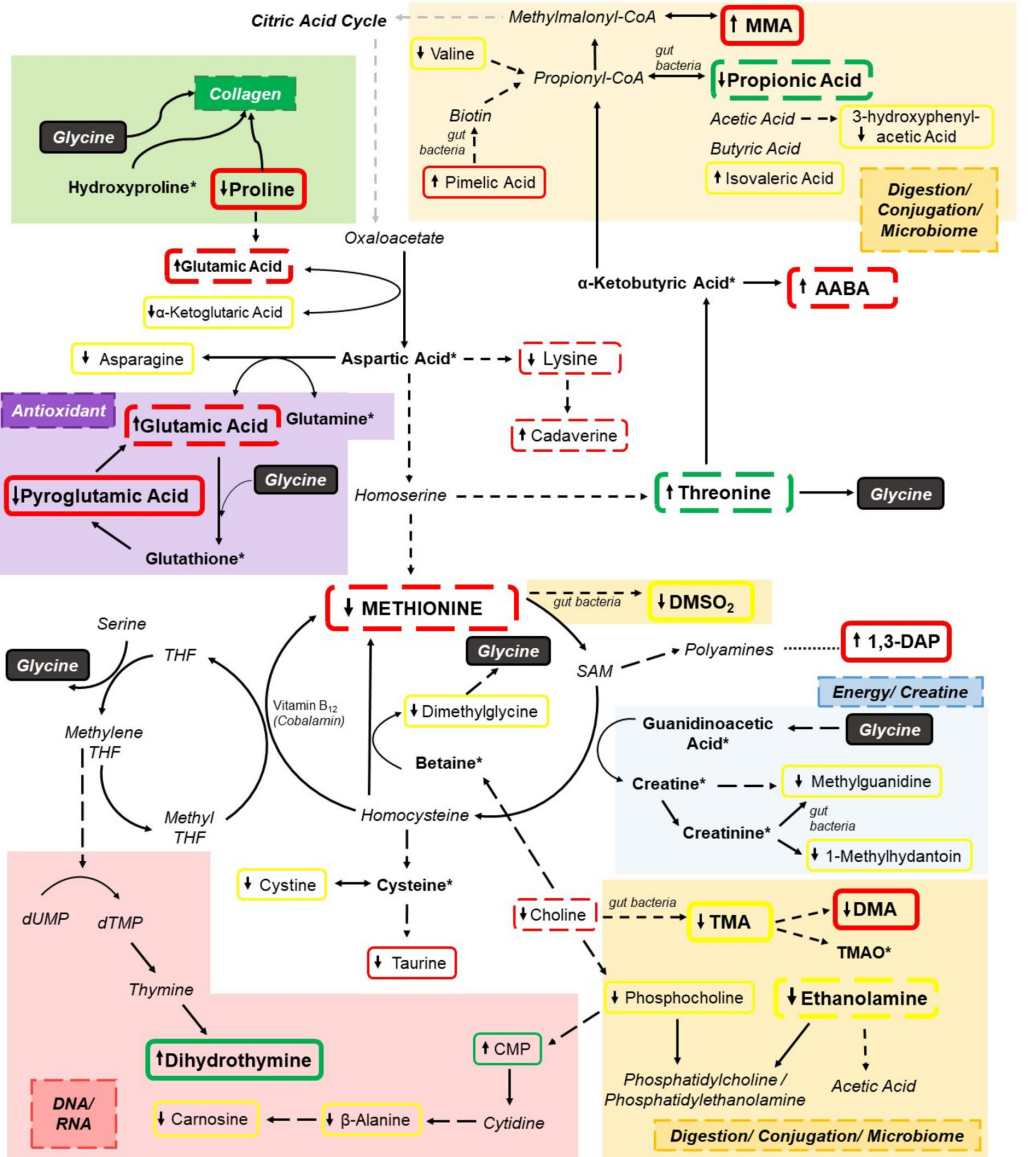
Pathways of the important metabolites identified in cheetahs. Green box represents glycine diet; red box represents baseline diet; yellow box represent baseline and glycine diet. Direction of box arrow indicates increase or decrease from control diet. Solid box indicates presence in urine, dotted box indicates presence in serum, italicised metabolites were not identified in this study; * indicates metabolites that were identified but similar across diets. Not all identified metabolites are included in this figure. MMA, methylmalonic acid; AABA, α-aminobutyric acid; DMSO_2_, dimethylsulphone;1,3-DAP,1,3-diaminopropane; SAM, S-adenosyl methionine; THF, tetrahydrofolate; TMP, thymidine monophosphate; UMP, uridine monophosphate; CMP, cytidine monophosphate

Seven serum metabolites were present in at least four out of the eight analyses and were, therefore, highlighted as important metabolites. These metabolites were L-glutamic acid, L-threonine, α-aminobutyric acid (AABA), glucose-6-phosphate (G-6-P), ethanolamine, L-methionine and propionic acid. None of the metabolites were significantly different between diets after FDR adjustment, but they still contributed to the variation observed between the diets. AABA, G-6-P and ethanolamine were identified in all 10 cheetahs across the three diets. However, the remaining metabolites were not consistently identified across diets. The relative concentration of the important metabolites found in the urine and serum across are highlighted in Table 4. The overall pathways identified are shown in Fig. 3.

**Table 4:** Important metabolites identified in the urine and serum of cheetahs at baseline, on a control diet and glycine-supplemented diet (*n* = 10)

Metabolites	Baseline	Control diet	Glycine-supplemented diet	Pathways involvement
URINE				
Dimethylsulphone	0.6 ± 0.1	1.0 ± 0.3	0.8 ± 0.1	Microbial origin. Production linked to methionine decomposition.
L-Proline	4.5 ± 0.4	5.5 ± 0.7	5.1 ± 0.5	Non-essential amino acid. Collagen biosynthesis.
D-Fructose	1.1 ± 0.3	*[low]	1.3 ± 0.5	Simple sugar. Endogenously produced from glucose and sorbitol.
Dimethylamine	0.7 ± 0.1	1.1 ± 0.2	0.9 ± 0.1	Organic amine. Biosynthesis mainly from TMA by gut microbiome.
Trimethylamine	0.1 ± 0.1	0.3 ± 0.1	0.1 ± 0.1	Organic amine. Biosynthesis from dietary choline, betaine, phosphatidylcholine and carnitine by gut microbiome.
Pyroglutamic acid	1.2 ± 0.3	1.7 ± 0.3	1.5 ± 0.3	Amino acid intermediate in glutathione cycle.
1,3-Diaminopropane	3.3 ± 0.3	3.0 ± 0.3	3.0 ± 0.3	Homologue of polyamine, putrescine. Unclear role. Potential link to ornithine, arginine availability.
Dihydrothymine	0.3 ± 0.0	0.4 ± 0.1	0.6 ± 0.3	Intermediate of thymine (pyrimidine) breakdown.
Methylmalonic acid	0.7 ± 0.2	0.6 ± 0.1	0.5 ± 0.2	Dicarboxylic acid intermediate in protein and fat metabolism.
Pimelic acid	0.7 ± 0.1	0.6 ± 0.1	0.6 ± 0.1	Precursor for biotin. Unclear pathway. Likely gut microbiome link.

SERUM				
L-Glutamic acid	0.9 ± 0.1	*[low]	*[low]	Abundant amino acid. Protein synthesis, glutathione production.
L- Threonine	0.5 ± 0.0	0.5 ± 0.1	0.6 ± 0.1	Essential amino acid. Lipid metabolism, epithelial mucin synthesis, maintaining intestinal health.
α-Aminobutyric acid	0.9 ± 0.1	0.8 ± 0.1	0.8 ± 0.1	Amino acid synthesised from methionine, threonine degradation. Unclear role.
Glucose-6-Phosphate	2.5 ± 0.3	2.3 ± 0.2	2.5 ± 0.2	Saccharide formed by phosphorylation of glucose. In all glucose linked pathways.
Ethanolamine	1.1 ± 0.1	1.2 ± 0.3	1.2 ± 0.2	In phospholipid cell membranes. Regulates lipid metabolism through gut microbiome.
L-Methionine	*[low]	0.5 ± 0.0	0.5 ± 0.0	Essential amino acid. Precursor for SAM (polyamine), homocysteine (folate cycle), succinyl CoA, phospholipids.
Propionic acid	0.5 ± 0.0	0.4 ± 0.0	*[low]	SCFA formed by fermentation of undigested starch/fibres by gut microbiome.

Data are expressed as relative concentration mean ± SD where applicable. *[low] indicates that the metabolite was detected in < 5 samples and, therefore, the overall relative concentration would be low and not fairly reflected with mean ± SD. SAM, S-adenosylmethionine; SCFA, short chain fatty acid

## Discussion

Ten important urinary metabolites and seven serum metabolites were identified in cheetahs at baseline, on a control diet and on a glycine-supplemented diet. These metabolites are involved in various pathways including energy metabolism, lipid metabolism, intestinal microbiome fermentation products, gastrointestinal health, glutathione production and pyrimidine biosynthesis (Table 4) (Brosnan & Brosnan, 2013; Dai et al., 2011; He & Slupsky, 2014; Tang et al., 2021; Wyss & Kaddurah-Daouk, 2000; Zhou et al., 2017). These pathways overlap with the diverse role of glycine and collagen and may indicate a dietary link in cheetahs. Specifically, the change in proline, pyroglutamic acid and 1,3-diaminopropane at baseline likely reflect the influence of a historic collagen diet and not necessarily glycine supplementation. Glycine supplementation had a similar effect to the baseline diet for DMSO_2_, TMA, DMA and pimelic acid– which indicates that glycine may influence the gut microbiome. Conversely, the effects of glycine supplementation exceeded the baseline diet for fructose, dihydrothymine and methylmalonic acid. Glutamic acid, AABA and methionine contributed primarily to the baseline diet and likely do not reflect specific changes to glycine supplementation. However, threonine, G-6-P, ethanolamine and propionic acid concentrations were influenced by glycine supplementation. The increased threonine and decreased propionic acid concentrations on the glycine-supplemented diet reflect changes in the gastrointestinal tract. There is notable overlap between the identified metabolites and three specific pathways: (1) methionine associated pathways, (2) threonine associated pathways and (3) glutamic acid associated pathways. Each of these are linked to specific physiological functions of glycine namely collagen biosynthesis, glutathione production, creatine and energy production, DNA and RNA biosynthesis, and digestion and conjugation as highlighted in Fig. 3 (Badenhorst et al., 2014; Li & Wu, 2018; Meléndez-Hevia et al., 2009).

### Methionine associated pathways

Methionine is an essential amino acid with a high dietary demand in cats, although cats are less tolerant of excess methionine than other amino acids indicating a high utilisation in the body (MacDonald et al., 1984). Previously methionine was identified at low concentrations relative to other amino acids in the urine and serum of cheetahs (Tordiffe & Mienie, 2019) as was found in the current study. Despite these low concentrations, methionine is clearly a hub of metabolism with many important metabolites identified in this study forming downstream substrates or products of methionine pathways. In terms of the gut microbiome, evidence suggests that DMSO_2_ production is linked to the decomposition of methionine by microbiota in the gastrointestinal tract and represents a major end product of methionine degradation (He & Slupsky, 2014). Therefore, the lower methionine concentration observed at baseline may have led to lower production and excretion of DMSO_2_ in the current study. Similarly, TMA (and subsequently DMA) concentrations were also lower, likely due to the lower concentration of its precursors choline and carnitine — betaine was identified, but not significantly different between groups. Interestingly, glycine supplementation also led to lower concentrations of DMSO_2_ and TMA despite methionine and choline only being lower at baseline. This may indicate that glycine supplementation is affecting the production of DMSO_2_ and TMA by reducing other sources (such as carnitine). Additionally, glycine in itself may be acting as a substrate leading to the reduced utilisation of other amino acids and producing different products, or that the bacterial population of collagen and glycine-rich diets are similar allowing the same bacteria to survive and produce similar products (Dai et al., 2011). Overall, the glycine diet appears to be providing the same gut bacterial effects as a more collagen-rich baseline diet.

The conversion of methionine to S-adenosylmethionine (SAM) is the major methionine catabolic pathway. The subsequent conversion of SAM to homocysteine allows the corresponding conversion of guanidinoacetic acid to creatine (Wyss & Kaddurah-Daouk, 2000). Glycine is directly involved in this process as the transfer of the amidino group of arginine to glycine yields ornithine and guanidinoacetic acid which represents the first step of creatine biosynthesis (Wyss & Kaddurah-Daouk, 2000). The reversible conversion of creatine to phosphocreatine via creatine kinase is crucial for rapid ATP production, particularly in cheetah muscles, both of which can spontaneously be degraded to creatinine (Kohn et al., 2024). Creatine and creatinine can also be converted by gut bacteria to methylguanidine, a uremic toxin, and along with other guanidino compounds (such as creatinine and creatine) could have neurotoxic effects if allowed to accumulate in the body. Similarly, 1-methylhydantoin can also be produced from the degradation of creatinine by gut bacteria and is either taken up by the body or degraded further by bacteria producing sarcosine which can lead to glycine biosynthesis (Wyss & Kaddurah-Daouk, 2000). Methylguanidine and 1-methylhydantoin were both significantly lower on the baseline and glycine diets compared to the control diet. This in itself may have positive health implications for the cheetah, but it may also indicate that more creatine is produced from the collagen and glycine-rich diets allowing the interconversion to phosphocreatine thereby producing more ATP. In the wild, cheetahs require rapid energy production to chase and capture prey, and this finding indicates that a collagen or glycine-rich diet (which would likely be reflected in their wild diet) may provide them with this crucial high energy phosphate to replenish ATP and also protect them from detrimental metabolites formed during the bacterial degradation of creatine and creatinine. Despite the lower physical activity experienced in captivity, these effects would undoubtedly still be beneficial for other metabolic processes.

Homocysteine, which is a key substrate produced from SAM, is involved in several important biological processes (Finkelstein, 1998). Firstly, homocysteine allows for the re-synthesis of methionine through the utilisation of betaine as a methyl donor forming dimethylglycine which ultimately forms glycine. The lower concentrations of dimethylglycine on the baseline and glycine diet indicates that this route is not utilised to the same extent when exogenous glycine is provided or when ingested collagen is high — the exact amount of glycine obtained from raw collagen in cheetahs is unknown but likely high. Additionally, choline concentrations were lower on the baseline diet indicating decreased catabolism to betaine, however on the glycine diet choline may be re-directed towards phosphocholine biosynthesis. Although none of the direct metabolites involved in the folate cycle were identified in the current study, homocysteine methylation also allows the recycling of 5-methyltetrahydrofolate (methyl-THF) to THF, leading to concomitant methionine production (Finkelstein, 1998). Crucially, THF to methylene-THF via glycine hydroxymethyltransferase allows the biosynthesis of glycine from serine– this is the major pathway for endogenous glycine biosynthesis in mammals accounting for 34 mmol/day glycine in humans (Meléndez-Hevia et al., 2009). Therefore, these two pathways highlight the close connection between glycine biosynthesis and the methionine cycle.

Owing to the lower concentrations of phosphocholine and ethanolamine, it appears that cheetahs on the collagen and glycine-rich diets have a lower source of phospholipids. This may also be due to the close link between lipid metabolism and the gut microbiome, in which the turnover of enterocytes and bacterial cells is a lipid-rich source that can be converted to ethanolamine and phosphatidylethanolamine (Zhou et al., 2017). The utilisation of ethanolamine by gut bacteria allows their survival and growth, therefore a lower concentration may indicate reduced bacterial colony growth and, along with the potential reduced biosynthesis of acetic acid, may indicate improved gastrointestinal health. Interestingly, CMP, which can be produced from phosphocholine and is a precursor for cytidine, had a higher urinary concentration in the glycine diet. This is echoed by the higher concentrations of dihydrothymine, a derivative of the pyrimidine, thymine, which was higher in the glycine diet and can be formed from the folate cycle using methylene-THF and, therefore, has a close relationship with glycine biosynthesis from serine as thymine biosynthesis leads to equimolar glycine production (Meléndez-Hevia et al., 2009). Although glycine is known to play a direct role in purine biosynthesis, this data also suggests a close relationship with pyrimidine biosynthesis as downstream products of glycine biosynthesis.

### Threonine associated pathways

Threonine is an essential amino acid in cats and its degradation is a minor source of glycine in humans (Meléndez-Hevia et al., 2009). The increased concentration of threonine on the glycine diet is likely due to the exogenous glycine reducing the amount of endogenous glycine required by threonine degradation, allowing the redirection of threonine elsewhere. This could lead to increased mucosal protein synthesis which accounts for approximately 70% of threonine utilisation (not depicted in Fig. 3) and would have intestinal benefits (Tang et al., 2021). It may also lead to increased flux towards α-ketobutyric acid and propionyl-CoA. Therefore, glycine supplementation may have a sparing effect on threonine, simulating dietary threonine supplementation shown to be beneficial in rodents, broilers and pigs (Tang et al., 2021).

At baseline, there is an increased conversion of α-ketobutyric acid to AABA which is not replicated by the glycine diet. Therefore, this would lead to increased production of propionyl-CoA on the glycine diet as indicated by the lower concentration of propionic acid. Similarly, the increased urinary methylmalonic acid concentrations at baseline (with glycine being significantly lower compared to baseline) indicates that glycine may be influencing the flux towards propionyl-CoA and methylmalonyl-CoA towards succinyl-CoA, and not necessarily the remnants of a collagen-rich diet. The increased concentration of pimelic acid on the baseline diet (and to a lesser extent on the glycine diet) may indicate a greater production of biotin, a coenzyme for propionyl-CoA carboxylase, that catalyses the conversion of propionyl-CoA to methylmalonyl-CoA (Zempleni et al., 2009). The reduced concentration of by-products of this pathway on the glycine diet may indicate greater flux towards the citric acid cycle. Conversely, the baseline diet may also be leading to greater flux through these pathways but is producing increased detrimental by-products such as methylmalonic acid and propionic acid. In terms of glycolysis, increased G-6-P on the baseline and glycine diet could then lead to increased pyruvate and increased acetyl-CoA production to enter the citric acid cycle. The slightly higher lactic acid on the glycine diet may indicate increased flux through glycolysis (producing lactic acid from pyruvate). The concentrations of pyruvate remained low and fairly constant between diets, supporting that any increase in glycolysis caused by glycine leading to pyruvate would be quickly converted to lactic acid or acetyl-CoA.

Propionic acid was the only SCFA identified in this study, likely due to the rapid enterocyte absorption or coenzyme A binding of SCFA for energy production (Zhou et al., 2017). Nonetheless, 3-hydroxyphenylacetic acid (Table 2) likely has a microbial origin as has been found with 4-hydroxyphenylacetic acid in cheetahs (Tordiffe et al., 2017). Cheetahs on a rabbit carcass diet produced lower concentrations of SCFA and putrefactive compounds, which the authors suggest is due to the beneficial effects of poorly digestible animal tissue such as hair and bones (Depauw et al., 2013). Although not significantly different, the current study identified increased propionic acid in the baseline diet with the glycine diet having the lowest identified relative concentration. This supports that it may be the poorly digestible animal tissue causing the beneficial SCFA effects and not necessarily any metabolic effects that a historic collagen-rich diet (as depicted by the baseline diet) may have. It also indicates that glycine may be eliciting a response as indicated by the apparent lower propionic acid concentration. Alternatively, the actual carcass tissue components may elicit the same response, but that would be over a shorter period and cannot be concluded from this study. Overall, considering that all cheetahs consumed a similar amount of muscle meat throughout the study, the reduced production of products of protein digestion and fermentation on the glycine diet compared to both the baseline and control diet, indicates a potentially positive gastrointestinal effect of glycine. This may be due to an increase in threonine allowing intestinal mucosa production, thereby improving intestinal integrity, as well as improved glycine conjugation of organic acids and other detrimental products formed during digestion and fermentation of the high protein diet consumed by captive cheetahs.

### Glutamic acid associated pathways

Glutamic acid had a high concentration on the baseline diet corresponding with the lower concentration of proline and α-ketoglutaric acid, indicating a greater conversion to glutamic acid. Glycine, proline and hydroxyproline collectively make up the vitally important structural protein, collagen (Li & Wu, 2018). Therefore, the lower urinary proline excretion on the baseline diet likely indicates a reduced turnover of collagen due to higher collagen ingestion and maintenance, while the opposite is seen on the collagen and glycine deficient control diet. The excretion of hydroxyproline was also slightly higher in the control group, although it was not identified as significant. Together, these indicate that the control group had a higher collagen turnover due to collagen deficiency, also found in young cheetahs with nutritional deficiencies (Tordiffe & Mienie, 2019). It is apparent that the baseline diet, still containing remnants of a collagen-rich diet, has the greatest influence and any impact that glycine supplementation may be having directly on collagen is minimal. It is interesting that despite the 3-week habituation period where no carcass components were consumed, there is still a clear metabolic influence of collagen that cannot be replicated by 4 weeks of glycine supplementation. This could be due to the collagen effect build up over a long period (cheetahs received mixed carcass components for at least 6 months prior to the study) or potentially the re-direction of glycine towards other physiological functions and not collagen biosynthesis. The latter could also emphasise that 30 g of glycine per kg meat is insufficient when the diet is completely collagen depleted. Additionally, this indicates that the baseline diet contains higher levels of glycine than would be expected.

Glutamic acid, glycine and cysteine are substrates required to produce the major antioxidant glutathione (Brosnan & Brosnan, 2013). Glycine and cysteine were similar across diets, while the higher glutamic acid concentration at baseline likely indicates a greater production of glutathione as reflected by the lower concentrations of pyroglutamic acid. This increased glutathione production would protect against oxidative damage and convey an improved immunity thereby explaining the higher eosinophil and basophil WBC count in the control and glycine groups (van Boom et al., 2024). Additionally, the lower pyroglutamic acid indicates that there is adequate glycine as they have an inverse relationship (Jackson et al., 1987). This supports the above theory that there was indeed a glycine deficiency on the control diet that was not fully corrected by glycine supplementation and may lead to a detrimental decreased biosynthesis of glutathione. Overall, it appears that collagen (or historic metabolic effects of collagen) has a greater impact on glutamic acid related pathways than glycine alone and that when glycine is limited, it appears to be redirected to other pathways in cheetahs, such as pathways that promote glycine biosynthesis.

### Study limitations and future directions

There were several limitations of this study, of which, the challenges pertaining to the study design have previously been reported (van Boom et al., 2024). The study was conducted with only 10 cheetahs and while this is reflective of the limited global captive population, a larger study cohort would yield a better representation of the metabolite data. Additionally, there were limitations associated with the NMR analysis and the ASICS software package. NMR inherently creates complex spectra in which it can be challenging to identify individual metabolites that are contributing to the spectra. The use of ASICS, as an automated software, was a simple and reliable approach to this challenge. However, it only contains a library of 190 metabolites and, therefore, only metabolites within this library could be identified in the current study. Moreover, the concentrations obtained were relative to the spectrum and not an absolute concentration. This has no direct implications to the data, but does limit comparison to the literature especially those using similar approaches in cats and cheetahs.

Future studies should investigate the metabolic response of wild or captive cheetahs to a whole carcass diet or specific individual components of the wild diet (such as collagen directly, skin, bones), as well as investigating these parameters in a population suffering from gastritis or other related health conditions. Additionally, it may be beneficial to use a targeted approach and alternative separation and identification techniques (such as GC-MS) to enhance the data obtained and focus on the key pathways and metabolites identified in the current study.

## Conclusion

This was the first study to investigate the metabolic effects of glycine supplementation in captive cheetahs to further understand nutrition and health in captivity. It appears that glycine supplementation had a direct effect on threonine- and methionine-related pathways, specifically that of threonine sparing, pyrimidine biosynthesis, and reduction of certain bacterial fermentation products which may prove to be beneficial in improving gastrointestinal health although no clear conclusions can be drawn from this study alone. Glycine supplementation had a limited effect on collagen and glutathione production, indicating that when cheetahs experience glycine or collagen deficiency, glycine is redirected to other pathways potentially those that promote glycine biosynthesis. Along with glycine, this study also highlighted the historic or remnant effect of a collagen-rich diet achieved through the consumption of bones, skin and connective tissue. This effect was similar to glycine supplementation for energy metabolism, specifically that of creatine production, carbohydrate and fat metabolism. These latter findings indicate that the effects of a collagen-rich diet can be maintained in cheetahs for up to three weeks even with no additional sources of glycine and collagen with depletion only occurring between three to seven weeks. Glycine supplementation may be a useful feeding tool to utilise in long periods when carcass diets and dietary collagen is limited or not readily consumed by cheetahs. The study findings highlight the diverse metabolic effects of glycine and collagen in captive cheetahs and future studies should use a targeted approach to further elaborate on these pathways.

## Electronic supplementary material

Below is the link to the electronic supplementary material.

Supplementary Material 1

## Data Availability

Data is provided within the manuscript or supplementary information files. Metabolomics and metadata reported in this paper are also available via: UP’s Research Data Repository (Figshare). Study identifier: 10.25403/UPresearchdata.24494851. The corresponding author may be contacted for further requests.
