# Supplementary material for: Methionine, threonine and glutamic acid adapted pathways in captive cheetahs on a glycine-supplemented diet
Source: Metabolomics. 2025 Apr 23;21(3):56. doi: 10.1007/s11306-025-02243-1 (PMC12018600; doi:10.1007/s11306-025-02243-1)
Supplement: Supplementary file 1 — Supplementary Material 1 [file 11306_2025_2243_MOESM1_ESM.docx]

**SUPPLEMENTARY TABLES**

**Title: Methionine, threonine and glutamic acid adapted pathways in captive cheetahs on a glycine- supplemented diet**

| **Table S1**. Nutrient breakdown of horse muscle meat, vitamin and mineral supplement and glycine supplement | | | |
| --- | --- | --- | --- |
| **Nutrient** | **Horse muscle meat (per 100g) ^a^** | **Vitamin and Mineral (per 10g) ^b^** | **Glycine (per 30g) ^b^** |
| **Moisture (g)** | 70.9 |  |  |
| **Protein (g)** | 19.8 |  |  |
| **Lipids (g)** | 6.63 |  |  |
| **Ash (g)** | 0.98 |  |  |
| **Cholesterol (mg)** | 61 |  |  |
| **Energy (kcal)** | 140 |  |  |
| **Short Chain FA (g)** | 2.0 |  |  |
| **Monounsaturated FA (g)** | 2.7 |  |  |
| **Polyunsaturated FA (g)** | 1.1 |  |  |
| Linoleic acid (mg) | 678 |  |  |
| α-linolenic acid (mg) | 318 |  |  |
| 20:2 n-6 (mg) | 19 |  |  |
| 20:3 n-6 (mg) | 14 |  |  |
| Arachidonic acid (mg) | 58 |  |  |
| **Ammonia (g)** | 0.26 |  |  |
|  |  |  |  |
| **Essential amino acids (g)** |  |  |  |
| Histidine | 0.90 |  |  |
| Isoleucine | 0.91 |  |  |
| Leucine | 1.52 |  |  |
| Lysine | 1.57 |  |  |
| Methionine | 0.48 |  |  |
| Phenylalanine | 0.82 |  |  |
| Threonine | 0.84 |  |  |
| Tryptophan | 0.15 |  |  |
| Tyrosine | 0.67 |  |  |
| Valine | 0.96 |  |  |
| **Non-essential/ Conditionally essential amino acids (g)** |  |  |  |
| Alanine | 1.18 |  |  |
| Arginine | 1.16 |  |  |
| Aspartic acid | 1.77 |  |  |
| Cystine | 0.20 |  |  |
| Glutamic acid | 2.83 |  |  |
| Glycine | 1.04 |  | 30 |
| Proline | 0.89 |  |  |
| Serine | 0.69 |  |  |
|  |  |  |  |
| **Hydroxyproline (g)** | 0.15 |  |  |
| **Total collagen (g)** | 1.17 |  |  |
| **Soluble collagen (%)** | 10.2 |  |  |
| **Purine bases (mg)** |  |  |  |
| **Adenine** | 18.29 |  |  |
| **Guanine** | 8.23 |  |  |
| **Xanthine** | 9.01 |  |  |
| **Hypoxanthine** | 74.03 |  |  |
|  |  |  |  |
| **Mineral (mg)** |  |  |  |
| Sodium | 74.2 |  |  |
| Potassium | 331.0 |  |  |
| Magnesium | 28.9 |  |  |
| Calcium | 3.8 | 3000 |  |
| Phosphorous | 231.0 | 670 |  |
| Iron | 3.9 |  |  |
| Zinc | 3.7 |  |  |
| Copper | 0.2 | 4.0 |  |
| Manganese |  | 1.5 |  |
| Sodium chloride |  | 340 |  |
|  |  |  |  |
| **Vitamins** |  |  |  |
| Vitamin A (IU) | — | 5000 |  |
| Thiamin (B_1_) (mg) | 0.043 | ­­­­­5.0 |  |
| Riboflavin (B_2_) (mg) | 0.18 | 2.5 |  |
| Niacin (B_3_) (mg) | 5.54 |  |  |
| Pantothenic acid (B_5_) (mg) | — | 10 |  |
| Pyridoxine (B_6_) (mg) | 0.64 |  |  |
| Biotin (B_7_) (µg) |  | 100 |  |
| Folic acid (B_9_) (µg) |  | 800 |  |
| Vitamin B_12_ (µg) | 2.08 |  |  |
| Vitamin C (mg) | — | 125 |  |
| Vitamin D_3_ (IU) |  | 100 |  |
| Vitamin E (mg) |  | 50 |  |
| *^a^ Values obtained from Badiani et al. (1997); ^b^ values obtained from WildCat Nutrition, Pretoria, South Africa* | | | |

| **Table S2.** The (A) PCA loadings, (B) PLS-DA VIP scores and (C) PLS-DA diet regression coefficients for the top 10 urinary metabolites in 10 captive cheetahs. | | | | | | | | |
| --- | --- | --- | --- | --- | --- | --- | --- | --- |
| 1. **PCA Metabolite loadings** | | | |  | 1. **PLS-DA Metabolite VIP Scores** | | | |
| **PC 1** | | **PC 2** | |  | **C 1** | | **C 2** | |
| Pantothenic acid | -0.17 | UDPG | -0.22 |  | Dihydrothymine | 2.63 | Dihydrothymine | 2.19 |
| L-Carnitine | 0.17 | Glutaconic acid | -0.21 |  | Methylmalonic acid | 2.40 | Methylmalonic acid | 1.79 |
| Malonic acid | 0.17 | Trimethylamine | 0.20 |  | Dimethylamine | 2.36 | Dimethylamine | 1.75 |
| Choline chloride | 0.16 | D-Fructose | -0.19 |  | Pyroglutamic acid | 2.13 | Pyroglutamic acid | 1.65 |
| Acetoacetic acid | 0.16 | Spermidine | 0.19 |  | L-Proline | 2.02 | GMP | 1.59 |
| L-Proline | 0.16 | Dimethylglycine | 0.19 |  | GMP | 1.94 | N-(2-Furoyl)Glycine | 1.57 |
| Isovaleric acid | -0.16 | Methylguanidine | 0.18 |  | 1,3-DAP | 1.93 | L-Proline | 1.55 |
| Acetaminophen | 0.15 | Lactic acid | -0.18 |  | D-Fucose | 1.89 | Adipic acid | 1.52 |
| Taurine | 0.15 | L-Histidine | -0.18 |  | Dimethyl sulphone | 1.69 | 1,3-DAP | 1.47 |
| Ethanolamine | 0.15 | 2-Deoxycytidine | 0.17 |  | L-Glutamine | 1.61 | UMP | 1.45 |

| 1. **PLS-DA metabolite coefficients for each diet** | | | | | |
| --- | --- | --- | --- | --- | --- |
| **Baseline** | | **Control** | | **Glycine** | |
| Dihydrothymine | 97.8 | Methylguanidine | 71.9 | Dihydrothymine | 100.0 |
| Dimethyl sulphone | 88.1 | Dimethyl sulphone | 68.1 | Dimethyl sulphone | 79.8 |
| Dimethylamine | 84.4 | Trimethylamine | 60.9 | N-(2-Furoyl)Glycine | 75.8 |
| N-(2-Furoyl)Glycine | 71.7 | Dimethylglycine | 56.2 | GMP | 74.1 |
| 1,3-DAP | 71.7 | α-Ketoglutaric acid | 52.1 | Pimelic acid | 68.3 |
| Methylmalonic acid | 71.2 | D-Fructose | 50.1 | Dimethylamine | 68.0 |
| GMP | 70.9 | 1-Methylhydantoin | 49.1 | Methylmalonic acid | 64.8 |
| Pimelic acid | 63.7 | D-Glucose | 46.9 | 1,3-DAP | 60.6 |
| L-Proline | 61.5 | Dimethylamine | 46.8 | Adipic acid | 59.4 |
| UMP | 60.4 | L-Valine | 44.6 | L-Tryptophane | 54.8 |
| *Loadings are the weight of each metabolite and are expressed as positive or negative number depending on its direction from the PC. Coefficients are the weighted sum of the PLS-DA regression for each group. PCA, principle component analysis; PLS-DA, partial least squares discriminant analysis, VIP, variable importance in projection; PC, principle component; C, component; TMAO, trimethylamine oxide; UDPG, uridine 5'-diphosphate glucose; GMP, guanosine monophosphate; UMP, uridine monophosphate; CMP, cytidine monophosphate; 1,3-DAP, 1,3-diaminopropane* | | | | | |

| **Table S3.** The (A) PCA loadings, (B) PLS-DA VIP scores and (C) PLS-DA diet regression coefficients for the top 10 serum metabolites in 10 captive cheetahs | | | | | | | | |
| --- | --- | --- | --- | --- | --- | --- | --- | --- |
| **A. PCA Metabolite loadings** | | | |  | **B. PLS-DA Metabolite VIP scores** | | | |
| **PC 1** | | **PC 2** | |  | **C 1** | | **C 2** | |
| L-Glycine | 0.25 | Lactic acid | 0.29 |  | L-Glutamic acid | 2.89 | L-Glutamic acid | 2.63 |
| Hypotaurine | 0.25 | L-Threonine | 0.28 |  | Propionic acid | 2.49 | Propionic acid | 2.12 |
| L-Carnitine | 0.24 | L-Valine | 0.28 |  | α -Aminobutyric acid | 1.94 | α-Aminobutyric acid | 1.65 |
| D-Glucose | 0.24 | L-Lysine | 0.28 |  | Lactose | 1.75 | Lactose | 1.54 |
| Guanidinoacetic acid | 0.24 | L-Glutamine | -0.27 |  | Choline chloride | 1.75 | Choline chloride | 1.50 |
| L-Cystine | 0.24 | Cadaverine | -0.25 |  | L-Threonine | 1.61 | L-Threonine | 1.36 |
| D-Glucuronic acid | 0.24 | Creatine | 0.24 |  | D-Mannose | 1.49 | D-Mannose | 1.34 |
| Ethanolamine | 0.23 | Creatinine | -0.24 |  | L-Methionine | 1.46 | L-Methionine | 1.30 |
| N-Acetylglycine | 0.22 | D-Gluconic acid | -0.22 |  | Lactic acid | 1.09 | Ethanolamine | 1.28 |
| Propylene glycol | 0.22 | Phenethylamine | -0.18 |  | L-Lysine | 1.02 | G-6-P | 1.02 |

| **C. PLS-DA metabolite coefficients for each diet** | | | | | |
| --- | --- | --- | --- | --- | --- |
| **Baseline** | | **Control** | | **Glycine** | |
| L-Glutamic acid | 100.0 | L-Glutamic acid | 47.6 | L-Glutamic acid | 32.6 |
| α-Aminobutyric acid | 66.1 | Taurine | 40.4 | Taurine | 23.6 |
| Propionic acid | 63.2 | Ethanolamine | 35.5 | G-6-P | 20.8 |
| Choline chloride | 55.8 | G-6-P | 35.2 | Ethanolamine | 20.3 |
| L-Methionine | 54.9 | L-Threonine | 34.3 | D-Maltose | 19.5 |
| Cadaverine | 44.8 | L-Valine | 34.1 | L-Threonine | 18.7 |
| Taurine | 44.5 | D-Maltose | 33.4 | L-Methionine | 17.8 |
| L-Lysine | 44.1 | Ascorbic acid | 29.0 | Propionic acid | 17.6 |
| D-Mannose | 43.3 | D-Galactose | 28.8 | L-Valine | 17.5 |
| G-6-P | 41.5 | D-Glucuronic acid | 26.6 | D-Galactose | 16.1 |
| *Loadings are the weight of each metabolite and are expressed as positive or negative number depending on its direction from the PC. Coefficients are the weighted sum of the PLS-DA regression for each group. PCA, principle component analysis; PLS-DA, partial least squares discriminant analysis, VIP, variable importance in projection; PC, principle component; C, component. G-6-P, D-Glucose-6-phosphate* | | | | | |
